# 

**Published:** 1884-05

**Authors:** 


					﻿iMJYY 1S84,
QUD SERIES
Vol/ XXV
-^Sl'S4£
& 1855 O
NEW SERIES
Vol. i -Hol N L


(JOURNAL
r	OV.o&su $
x^bv1
1 W.F. WESTMOREL AND, Mil
H.V.M.MILLER, M.D.LL ,D.i
JAMES A.GRAY, M.D.
0UBUSHED KY JAMES P.HARRISON&CQ?
c	Atlanta,ga.
SUBSCRIPTION: 2.50 A YEAR.
				

